# Graphene-Oxide Peptide-Containing Materials for Biomedical Applications

**DOI:** 10.3390/ijms251810174

**Published:** 2024-09-22

**Authors:** Andreea Gostaviceanu, Simona Gavrilaş, Lucian Copolovici, Dana Maria Copolovici

**Affiliations:** 1 Institute for Interdisciplinary Research, Aurel Vlaicu University of Arad, Elena Drăgoi St., No. 2, 310330 Arad, Romania; andreea.andreea010@yahoo.com (A.G.); simona.gavrilas@uav.ro (S.G.); lucian.copolovici@uav.ro (L.C.); 2 Biomedical Sciences Doctoral School, University of Oradea, University St., No. 1, 410087 Oradea, Romania; 3 Faculty of Food Engineering, Tourism and Environmental Protection, Aurel Vlaicu University of Arad, Elena Drăgoi St., No. 2, 310330 Arad, Romania

**Keywords:** graphene oxide, peptides, proteins, graphene, applications, biomedicine, antibacterial, cancer

## Abstract

This review explores the application of graphene-based materials (GBMs) in biomedicine, focusing on graphene oxide (GO) and its interactions with peptides and proteins. GO, a versatile nanomaterial with oxygen-containing functional groups, holds significant potential for biomedical applications but faces challenges related to toxicity and environmental impact. Peptides and proteins can be functionalized on GO surfaces through various methods, including non-covalent interactions such as π–π stacking, electrostatic forces, hydrophobic interactions, hydrogen bonding, and van der Waals forces, as well as covalent bonding through reactions involving amide bond formation, esterification, thiol chemistry, and click chemistry. These approaches enhance GO’s functionality in several key areas: biosensing for sensitive biomarker detection, theranostic imaging that integrates diagnostics and therapy for real-time treatment monitoring, and targeted cancer therapy where GO can deliver drugs directly to tumor sites while being tracked by imaging techniques like MRI and photoacoustic imaging. Additionally, GO-based scaffolds are advancing tissue engineering and aiding tissues’ bone, muscle, and nerve tissue regeneration, while their antimicrobial properties are improving infection-resistant medical devices. Despite its potential, addressing challenges related to stability and scalability is essential to fully harness the benefits of GBMs in healthcare.

## 1. Introduction

Graphene-based materials (GBMs), including graphene (G) and graphene oxide (GO), are renowned for their exceptional properties, such as strength, conductivity, and flexibility. Graphene’s single-layer honeycomb structure contrasts with GO, which features oxygen functional groups that enhance water dispersibility and scalability. These attributes make GBMs highly versatile for applications across various biomedical fields, including biosensors, drug-delivery systems, biomedical imaging, tissue engineering, and antimicrobial medical devices [1,2].

The functionalization of GO with peptides has emerged as a crucial strategy to enhance its biocompatibility and functionality. Previous reviews have thoroughly examined the integration of GO with peptides and proteins, highlighting significant progress [3,4,5]. For instance, antimicrobial peptides (AMPs) like HHC-36 [6] and CATH-2 [7] have been combined with GO to boost antimicrobial activity, while cell-penetrating peptides (CPPs) such as Octaarginine (R8) [8] improve gene and drug delivery efficiency. These reviews also discuss the broad applications of GO–peptide conjugates, including their roles in biosensors for enhanced sensitivity and specificity, medical devices and implants leveraging GO’s antimicrobial properties, tissue engineering for supporting cell growth and tissue regeneration, and biomedical imaging where GO enhances contrast and imaging precision.

Despite these advances, there are still gaps in understanding the full spectrum of peptide–GO interactions and their long-term effects [9]. This review provides a comprehensive overview of recent advancements and explores new functionalization strategies for GO–peptide conjugates [10], emphasizing the need for ongoing research to address safety and efficacy concerns.

## 2. Understanding GO and Its Derivatives

Graphene (G) and graphene oxide (GO) are key materials with exceptional properties. They hold significant promise for numerous applications, including biomedicine [11].

**Figure 1:**
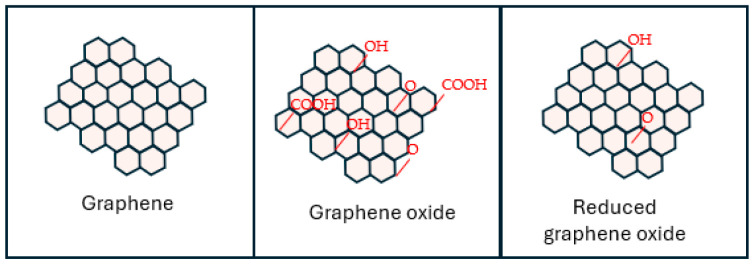
Structural representations of graphene, graphene oxide, and reduced graphene oxide. Based on Figure 1 from Fallahazad, Rational and key strategies toward enhancing the performance of graphene/silicon solar cells, 2023 [17].

They are part of the graphene-based materials family (GBMs), along with reduced graphene oxide (rGO) (Figure 1), bilayer graphene, multilayer graphene, and chemically modified graphene [12,13,14]. GBMs vary in oxygen content, number of layers, surface chemistry, purity, lateral dimensions, defect density, and composition [15]. G and its derivatives exhibit remarkable versatility, making them promising candidates for significant biomedical applications [16]. 

Graphene (G) is a single-atom-thick sheet of hexagonally organized *sp*^2^-bonded carbon atoms. It has sparked intense interest in the last decade due to its exceptional mechanical, electronic, optical, and chemical properties [11]. As the strongest and thinnest nanomaterial known, G’s attributes include a large surface area and high permeability, which are advantageous for various applications [1,18].

Graphene oxide (GO), a two-dimensional nanomaterial composed of a single-atom-thick layer of graphene sheets [15], is formed through the chemical oxidation of natural graphite at various levels [19]. After oxidation, GO can disperse in water due to the presence of hydroxyl (–OH), carboxyl (–COOH), and epoxide (C–O–C) groups. Carboxyl groups provide stability and negative charge, while hydroxyl and epoxide groups enable biomolecule functionalization, aiding bio-applications [11]. GO has several advantages over G, including easy processing, large-scale synthesis, and low production costs. It is frequently used as a starting point to make rGO in efforts to restore its *sp*^2^ hybridization structure [1,11]. Though hydrazine and similar reducing agents are toxic, recent studies have developed less harmful protocols using ascorbic acid to produce rGO with higher biocompatibility. However, these methods only partially remove oxygen species from GO, leaving rGO with limited oxidized functional groups needed for aqueous suspension [11]. Significant advancements have been made in functionalizing GO. It has been used for desalination, drug delivery, oil–water separation, immobilization catalysis, solar cells, energy storage, and healthcare, among other things [1,15].

Graphene-based materials (GBMs) are typically classified based on three properties: the number of layers, average lateral dimension, and carbon/oxygen ratio [20]. Single-layer G is characterized by an extended honeycomb network, which serves as the fundamental structural component of other significant allotropes. G can be stacked to create three-dimensional graphite, rolled to create one-dimensional carbon nanotubes (CNTs), and wrapped to create zero-dimensional fullerenes [15].

Regarding average lateral dimension, the size of graphene sheets can vary, which impacts their application and functionality. The carbon/oxygen ratio is crucial, as it affects the material’s chemical properties and usability. G has several advantages over carbon nanotubes (CNTs). For instance, the material’s enhanced permeability and retention (EPR) effect facilitates its uptake by cells and retention in tumors, reducing its cytotoxicity and improving its photothermal sensitivity. Additionally, its larger surface area and susceptibility to molecular modification facilitate various potential applications [21]. A notable advancement in this area is PEGylated nanographene, which incorporates branched polyethylene glycol to enhance cellular absorption and tumor retention [15].

With the growing use of nanotechnology, human and environmental exposure to graphene-based nanomaterials is expected to rise, making a comprehensive understanding of GBM toxicity crucial for their potential biomedical applications [22]. Given the possible risks associated with their manufacture and use, over the past ten years, there has been a sharp increase in nanotoxicological studies conducted on graphene-related materials. These studies explore the biological and nanostructural interactions at various levels of living systems, from biomolecules to whole organisms [16].

While the precise structure of GO remains a topic of constant discussion, it is widely accepted that the presence of epoxide, hydroxyl, carbonyl, and carboxylic groups disrupts the aromatic framework of G. The prevailing model, proposed by Lerf and Klinowski [23], suggests that heavily oxygenated GO consists of hydroxyl and epoxide functional groups on the basal planes and carbonyl and carboxyl groups at the edges. Oxygen functionalities make the GO layers hydrophilic, allowing water molecules to insert themselves between them easily [5].

## 3. Methods for Synthesizing GO

GO and other GBMs can be synthesized using various methods, which are generally categorized into “bottom-up” and “top-down” approaches [24]. The “bottom-up” methods involve the treatment of small molecular precursors such as citrate [25] and saccharide [26] acid via combustion or solvothermal processes. In contrast, “top-down” methods break down larger carbon sources, including graphite, carbon nanotubes, and Platanus leaves, through techniques like electrochemical oxidation, arc discharge, and laser ablation [24,27].

Historically, GO was first synthesized by Brodie in 1859 using KClO_3_ and HNO_3_ with graphite [28]. Staudenmaier modified this protocol in 1898 by adding HClO_3_ gradually to a mixture of concentrated H_2_SO_4_ and fuming HNO_3_ [29]. This minor modification made highly oxidized GO production easy. However, Brodie’s and Staudenmaier’s methods generated harmful gases and were time-consuming [1].

The widely recognized Hummers’ method [30], introduced in 1958, uses KMnO_4_ NaNO_3_ in concentrated H_2_SO_4_. While effective, this method produces heavy metal pollution and challenges in removing by-products like Na^+^ and NO_3_^−^ ions [31]. Marcano’s method [32], using less corrosive H_3_PO_4_, offered higher oxidation and structural regularity but increased costs and waste treatment. Other methods, like K_2_FeO_4_/H_2_SO_4_ or benzoyl peroxide (C_14_H_10_O_4_), were safe and efficient but had drawbacks like high material costs and instability. Raw materials and reaction methods also influenced GO properties, with higher crystalline graphite yielding fewer functional groups [19]. Modifying Hummers’ methods with ultrasound assistance [33] increased layer spacing but resulted in excessive oxidation and instability at high temperatures [1].

**Figure 2:**
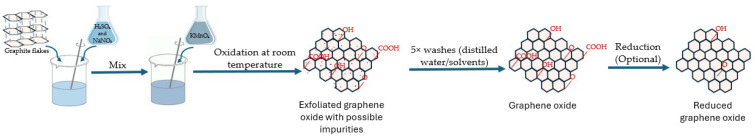
Overview of the Hummers’ method for synthesizing graphene oxide. Based on the text from [34].

Overall, each method for synthesizing graphene oxide (GO) has unique considerations and implications [24]. The choice of method depends on the desired properties of the GO, scalability, and specific application requirements. Among these methods, the Hummers’ method (Figure 2) is particularly favored for its simplicity and scalability. The Hummers’ method proceeds as follows [30,34]:

- Oxidation of graphite:
  - Graphite is mixed with concentrated H_2_SO_4_ and NaNO_3_.
  - KMnO_4_ is added slowly to the mixture while stirring.
- Exfoliation and expansion:
  - The resulting mixture is stirred and maintained at a specific temperature to initiate the oxidation of graphite.

This leads to the formation of GO, which has oxygen-containing functional groups on the surface, such as epoxide, hydroxyl, and carboxyl groups.

- Purification:

The exfoliated GO is repeatedly washed with water or other solvents to remove any residual acidic or oxidizing agents and by-products of the oxidation process.

- Optional reduction:
  - GO obtained from the previous steps may undergo reduction to remove some oxygen-containing functional groups and restore *sp^2^* carbon–carbon bonds, resulting in rGO.
  - This reduction can be achieved through various methods, such as chemical reduction using hydrazine or thermal treatment.

Lately, the modified Hummers’ method has gained recognition owing to its enhancements like improved efficiency, higher yield, and tailored material properties. To optimize the synthesis process, the modified Hummers’ method typically involves adjustments to the reaction conditions, such as temperature, reaction time, or the concentration of reagents [35]. Additionally, alternative oxidizing agents (KMnO_4_, NaNO_3_, and H_2_O_2_) or reducing agents (N_2_H_4_·H_2_O, Vitamin C, NaBH_4_, and a mixture of biomolecules from plant extracts) may be used to tailor the properties of the resulting GO or rGO materials [36].

Taking these methods further, Liu et al. [24] proposed a one-pot solvothermal method for selectively synthesizing pure GO or graphene oxide quantum dots (GOQDs). In this approach, graphite is used as the starting material and mixed with KMnO_4_ and concentrated H_2_SO_4_, followed by the gradual addition of HNO_3_. The reaction is carried out in a sealed autoclave, simplifying the operational procedure and creating high pressure, leading to enhanced oxidation. Adjusting the reaction temperature within the autoclave—from 130 °C to 170 °C—enables precise control over the product, yielding either GO or GOQDs of various sizes. GOQDs as small as 2.5 nm, with strong luminescence properties, are obtained at higher temperatures. This method is versatile and scalable, allowing for the use of multiple carbon sources. The size of the GOQDs can be further tuned by varying the graphite-to-KMnO_4_ ratio, making this process adaptable for large-scale synthesis and a wide range of applications [24].

## 4. Structural and Property Analyses of GO Derivatives

Given the increasing interest in using GBMs for medical applications, the issue of biocompatibility has become more crucial. GBMs’ biocompatibility is influenced by their intrinsic physicochemical properties [37], which will be discussed further below.

G and GO exhibit remarkable electrical, mechanical, and thermal properties due to their unique structures [38]. They demonstrate the Hall effect, tunneling effect, bipolar electric field effect, and high thermal conductivity. GO has a polydisperse sheet size and numerous oxygen-containing functional groups, making its structure more complex and property-dependent [1]. Lerf and Klinowski [23] proposed the L–K model, illustrating a random distribution of hydroxyl and epoxy groups, with carboxyl and carbonyl groups at the edges. GO’s aromatic (*sp*^2^) and aliphatic (*sp*^3^) domains increase surface interactions. Although GO can be reduced to G, the resultant G may not be suitable for specific applications due to structural defects. Nonetheless, this reduction process remains preferable for large-scale modification of graphene material properties through functionalization [1].

One of the main difficulties in characterizing GMBs is accurately identifying and quantifying the number of G layers present. This is mainly because, among ensembles of thicker crystals, monolayers are frequently in the extreme minority. Various techniques can be considered for analyzing G and its derivative materials. Commonly used techniques include high-resolution transmission electron microscopy (HRTEM), Raman spectroscopy, X-ray diffraction (XRD), atomic force microscopy (AFM), scanning tunneling microscopy (STM), and nitrogen adsorption–desorption at 77 K [39]. In order to accurately analyze the morphology, texture, crystal structure, and intrinsic properties of GMBs, it is essential to use two or more techniques, as no single technique can provide all the required information [18].

High-resolution transmission electron microscopy (HRTEM) can be used to investigate the atomic structure of both GO and G. This technique is crucial, as it enables the assessment of the crystalline nature of G flakes by analyzing their electron diffraction patterns [40]. On TEM images, the central region of the sheets typically appears as smooth and unblemished areas, with wrinkles that can be identified as dark marks. However, towards the edges, the sheets tend to curl. These folds exhibit a distinct TEM signature that indicates the number of layers in the G [18].

Raman spectroscopy is a robust and non-destructive method for analyzing carbon-based materials, especially when determining the difference between ordered carbon crystals and those not ordered. Consequently, it offers a rapid and effortless method to assess the composition and quality of GO and G [41]. Just like TEM analysis, Raman spectroscopy can be performed to ascertain the quantity of G layers present. This technique is exceptionally responsive to a small number of layers. As a result, it is possible to effectively differentiate between a single layer, a bilayer, and a few layers (fewer than five). With more than five layers, the Raman spectra appear nearly indistinguishable from those of bulk G [18].

X-ray diffraction (XRD) can be used to analyze the crystalline structures of both pure G and GO. The diffraction peak observed in both G and exfoliated GO is caused by the AB stacking arrangement, precisely the (002) reflection. The peak is observed at an angle of approximately 26° (2θ) for pristine graphite, but the same peak is shifted to an angle of roughly 11° (2θ) after the oxidation of the layers [18]. The interlayer distance, also known as d-spacing, for graphite, is commonly 0.335 nm. The oxidation of graphite leads to an increase in the d-spacing, which suggests the existence of intercalated species located between the layers of G [42]. A narrow XRD pattern peak indicates a high layer concentration in the sample [18].

Atomic force microscopy (AFM) is the most prominent technique for identifying individual and sparse-layered crystals [43]. While scanning the surface, the cantilever’s tip can analyze the sample’s topography and count layers by measuring differential height at the edge. Various AFM techniques enable the investigation of G flakes’ mechanical, electrical, magnetic, and elastic characteristics [18]. AFM can directly measure the number of layers [43], whereas scanning tunneling microscopy (STM) images can reveal G’s morphology and defects. This method allows single-layered G to be viewed in atomically resolved images [18].

Except for scanning tunneling microscopy (STM), all of the other examples of local analysis techniques described up until this point can approximate the number of layers present in a GO sample. Although this is not the case for gas adsorption measurements (typically N_2_ adsorption–desorption at 77 K), these measurements can potentially provide beneficial information regarding the textural characteristics of bulk samples [32].

By utilizing the analytical methods described above, we can gain valuable insights into the properties of GO and its derivatives. Below, we delve into the specific properties revealed by these analytical techniques.

Graphene-based materials derive their negative surface charge and colloidal stability from their peripheral carboxylate group, which depends on pH. The epoxide (–O–) and hydroxyl (–OH) groups on the basal plane are polar but uncharged, enabling weak interactions, hydrogen bonding, and other surface reactions [11]. Also, hydrophobic free surface π-electrons are present in unmodified G regions on the basal plane. These electrons can engage in π–π interactions, making them suitable for drug loading and non-covalent functionalization [44]. GO is a molecule with a sheet-like structure that exhibits amphiphilic properties. It can be employed as a surfactant to enhance the stability of hydrophobic molecules in aqueous solutions. Furthermore, due to functional groups, GO exhibits a significant defect density, resulting in diminished mechanical, electrical, and thermal characteristics compared to G or rGO [15].

rGO can be obtained from GO in a reducing environment, such as hydrazine or other reducing agents [45]. GO and rGO exhibit numerous disparities, particularly in their structural characteristics. GO has a higher concentration of oxygen-containing functional groups compared to rGO. The crystal structure of rGO is superior to that of GO [46]. Their structural variations result in distinct physicochemical characteristics. For instance, rGO exhibits superior optical absorbance and electrical conductivity compared to GO, although its hydrophilicity and surface charge are lower than GO [15].

G possesses a flawless plane and is almost entirely devoid of oxygen groups, resulting in exceptional electrical and optical conductivity. Although GO possesses numerous oxygen-containing groups, it exhibits limited electrical and optical conductivity [44].

Essentially, the electrical conductivity of rGO is superior to that of GO but inferior to that of G. Regarding water solubility, rGO is superior to G but inferior to GO due to variations in the amount of oxygen-containing groups present in each material. GO exhibits hydrophilic properties. In contrast, G is hydrophobic and does not disperse well in water. Regarding reactivity, rGO exhibits lower reactivity than GO, but it is still superior to G [15].

## 5. Functionalization of GO Derivatives with Peptides and Proteins

**Figure 3:**
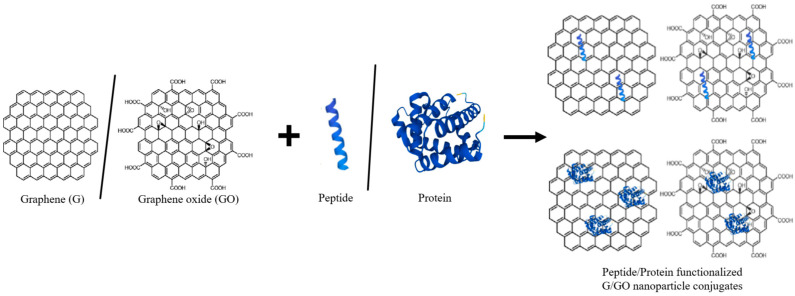
Functionalization of G/GO with peptides/proteins for nanoparticle conjugates (reproduced from [47] with permission from Elsevier, license number 5800301004281).

Functionalized GOs, including rGO, GO nanoplates, and sulfonated graphene oxide (sGO), exhibit distinct physical morphologies that influence membrane geometries and separation performance. Additionally, G/GO can be further functionalized with peptides or proteins (Figure 3, Table 1) to enhance its biocompatibility and functionality for various biomedical applications [19].

### 5.1. Peptides for Functionalizing GO

As an alternative to conventional methods for synthesizing GBMs, bio-inspired approaches have been explored to identify peptides with affinity for 2D nanomaterials, including G and GO. These peptides adhere to the surface by forming a complex binding pattern with the residues (usually 12) in their sequence [6].

Ou et al. [48] observed that α-helical peptides adsorbing to the G surface unravel and form amorphous dimers with minimal β-sheet formation. Kawazoe et al. [49] conceptualized the interaction of G and carbon nanotubes with phenylalanine (Phe), histidine (His), tyrosine (Tyr), and tryptophan (Trp) molecules. Results indicate that amino-acid aromatic rings align parallel to G and carbon nanotubes via π–π interactions, with Trp having higher affinity than Tyr, Phe, and His. Meanwhile, Ye et al. [50] conducted an experimental investigation into the interactions between GO and His, lysine (Lys), arginine (Arg), Trp, Tyr, and Phe. They showed that amino acids bound to the GO surface in the order of Arg > His > Lys > Trp > Tyr > Phe. These findings indicate that similar to protein adsorption, short-chain peptide adsorption on the GO is caused by multifunctional interactions between peptide molecules and the GO [51].

The conjugation of antimicrobial peptides (AMPs) with nanoparticles has been proposed to increase the peptide’s local concentration at the site of delivery, thus increasing efficacy [52].

**Table 1:** Peptide/Protein functionalized G/GO nanoparticle conjugates and their functional behavior.

Peptide/ Protein	Sequence	Peptide/Protein Binding Target:	Functional Behavior	References
HHC-36	KRWWKWWRR	G/GO	Demonstrates antimicrobial activity when functionalized with graphene.	[53]
CATH-2	RFGRFLRKIRRFRPKVTITIQGSARF	G/GO	Enhances antimicrobial properties when functionalized with G/GO.	[52]
Octaarginine (R8)	RRRRRRRR	G/GO	Evaluated for efficient gene delivery with GO.	[54]
Chlorotoxin	MCMPCFTTDHQMARKCDDCCGGKGRGKCYGPQCLCR	G/GO	Used for targeted glioma therapy with GO.	[55]
RGD2	RGD	G/GO	Improves efficiency of chemotherapeutic agents with GO.	[56]
GAMHLP-WHMGTL	GAMHLPWHMGTL	G/GO	Facilitates the separation and stabilization of graphene sheets.	[51]
P1 peptide	HSSYWYAFNNKT	G/GO	Used for exfoliating graphene and functionalizing it for biosensing applications.	[4]
GR3R	GGGGAAGGGGRRR	G/GO	Forms stable layers on graphene for molecular film creation and surface interaction studies.	[57]
Melittin	GIGAVLKVLTTGLPALISWIKRKRQQ	G/GO	Enhances antimicrobial efficacy of graphene-based composites.	[58]
LL-37	LLGDFFRKSKEKIGKEFKRIVQRIKDFLRNLVPRTES	G/GO	Exhibits antimicrobial properties for wound healing with G/GO.	[59]
Magainin	GIGKFLHSAKKFGKAFVGEIMKS	G/GO	Shows antimicrobial activity in GO-functionalized materials.	[60]
β-amyloid peptide	DAEFRHDSGYEVHHQKLVFFAEDVGSNKGAIIGLMVGGVVIA	G/GO	Studied for amyloid aggregation and Alzheimer’s intervention with GO.	[61]
α-synuclein	MDVFMKGLSKAKEGVVAAEKTKQGVAEAAGKTKEGVLYVGSKTREGVVQGVVTGVTAVA	G/GO	Studied for neurotoxic effects and therapeutic interventions with graphene.	[62]
β-defensin 2	GIGDPVTCLKSGAICHPVFCPRRYKQIGTCGLPGTKCCKKP	G/GO	Enhances antimicrobial properties in G/GO-based surfaces and is effective in bone reconstruction.	[63]
Bovine serum albumin (BSA)	583 amino acids	G/GO	Enhanced stability and functionality are potentially useful for biomedical applications such as biosensing and drug delivery.	[64]
Lysozyme	129 amino acids	G/GO	Preservation of enzymatic activity, facilitating its use in various industrial and biomedical applications, including antimicrobial coatings and biosensors.	[65]
Hemoglobin	Α-chain: 141 amino acids B-chain: 146 amino acids	G/GO	Oxygen-carrying capabilities, making it suitable for applications such as artificial blood substitutes and biosensors.	[66]
Streptavidin	159 amino acids	G/GO	Enhanced sensitivity and specificity in biosensing applications, facilitating the detection of various biomolecules.	[67]
Fibronectin	Several hundred to over a thousand amino acids	G/GO	Promotion of cell adhesion and growth, enhancing tissue regeneration for wound healing and regenerative medicine applications.	[68]
Pepsin A	326 amino acids	G/GO	Preservation of enzymatic activity and stability, enabling its use in biocatalysis and enzymatic systems for protein digestion and pharmaceutical manufacturing.	[69]
Myoglobin	153 amino acids	G/GO	Enhanced electrochemical performance in biosensing and bioelectronic devices, particularly for detecting oxygen levels and monitoring physiological conditions.	[70]

Peptide/Protein sequences were determined using Protein Data Bank (https://www.rcsb.org/ (access date: 25 July 2024)) [71]. Researchers have found that the AMP HHC-36, a 9-amino acid peptide with high charge density [72], can potentially adsorb to GO and form GO-HHC-36. The same study showed that GO-HHC-36 exhibits good biocompatibility with mesenchymal stem cells (MSCs), promoting their adhesion, migration, and differentiation [53].

Chicken cathelicidin-2 (CATH-2), a 26-amino acid peptide with high cationic and amphipathic properties, acts as a broad-spectrum AMP, serving as a model for developing novel antimicrobial agents [7]. Functionalizing GO with this peptide could significantly enhance its potential by leveraging CATH-2’s antimicrobial activity and GO’s unique physicochemical properties, paving the way for the development of highly effective antimicrobial materials with diverse biomedical applications. The resulting material, known as CATH-2-functionalized GO or GO/CATH-2, represents a promising advancement in the field of antimicrobial materials [73].

There has also been increasing research interest in cell-penetrating peptides (CPPs) due to their potential to enhance nanoparticle-mediated gene and drug delivery [32].

Octaarginine (R8), an 8-amino acid CPP particularly notable for its efficient cellular uptake properties, has been attached to GO and evaluated as a gene delivery carrier. R8 peptide is linked to carboxylated GO in a two-step amidation process at different ratios (0.1–1.5 μmol per mg of GO) [8]. Furthermore, Wang et al. proved the successful development of a novel delivery carrier, GO-R8/anti-HER2 (GRH), through conjugating R8 and anti-HER2 antibody with GO as a tumor active-targeting vector for survivin–siRNA delivery [74].

Chlorotoxin, a 36-amino acid peptide from the giant Israeli scorpion’s (*Leiurus quinquestriatus*) venom, has received attention for its high selectivity for glioma and other neuroectodermal tumors by binding to matrix metalloproteinase-2. Wang et al. [75] synthesized targeted peptide chlorotoxin-conjugated GO (CTX-GO) sheets that can be loaded with the chemotherapy drug Doxorubicin (DOX). Their study showed that, compared to GO loaded with Doxorubicin alone or free Doxorubicin, CTX-GO/DOX mediated the highest rate of glioma cell death [55].

Tumor-homing peptides (THPs), a subtype of CPPs, are extensively studied for their ability to bind specifically to tumor cells or their microenvironment [76].

An example is the RGD2 motif, found in extracellular matrix components like fibronectin and vitronectin, with high binding affinity to integrins. RGD analogs are applied in tumor imaging, anti-angiogenesis, and targeting with radionucleotides or chemotherapy [77]. Research demonstrates the use of RGD for functionalizing GO, which can then be utilized for loading DOX, enhancing delivery efficiency in hepatocellular carcinoma (HCC) treatment [56].

Katoch et al. [78] demonstrated that a dodecamer peptide, GAMHLP-WHMGTL, can adsorb onto the G surface, forming a complex reticular structure. This binding pattern can be applied to cause the separation of nanosheets in the case of G. An example of exfoliating G has been shown by using the G-binding P1 peptide (HSSYWYAFNNKT) with low sonication conditions. Sonication is used to supply enough energy to separate the individual sheets in the bulk graphite. This separation occurs because peptides adhere to the exposed surfaces, causing exfoliation [79]. Remarkably, employing this bio-based method leads to the extraction of G sheets with reduced levels of defect integration. The P1 peptide not only facilitates G exfoliation but also allows for adding extra features onto the G surface. To achieve this objective, the peptide can undergo chemical modification to introduce additional functional groups into its sequence [4]. Moreover, recent findings show that a mixture of P1 peptides and GR3R peptides (consisting of (GA)_3_ with arginine at both ends and three additional glycines at the *N*-terminus) at various ratios form a monomolecular-thick peptide layer on G. Because of this, the ordered molecular films’ self-assembled structures remain stable in liquid conditions [80].

Peptides can be functionalized onto the G surface either covalently or non-covalently. Peptides are carefully chosen or designed to act as the molecular analogs that interact with G or GO [77]. They can be customized to perform specific functions, such as improving compatibility with living organisms, enabling precise delivery of drugs to targeted areas, or possessing inherent therapeutic properties. The desired properties of the ultimate nanostructure determine the selection of peptide sequence, length, and modifications. Peptides that contain a high number of aromatic or hydrophobic residues (e.g., melittin, LL-37, magainin, β-amyloid peptide, α-synuclein, and β-defensin) can facilitate strong interactions with the G surface, thereby assisting in the process of self-assembly [5].

### 5.2. Proteins for Functionalizing GO

Recent studies indicate that GO–protein conjugates offer advantages over free proteins, including unique biological and chemical properties, enhanced biocompatibility and stability in biological environments, receptor or site targeting, and cellular internalization [11].

Proteins are indispensable for biological processes and regenerative therapies. Due to their biocompatibility, biodegradability, and bioresorbability, they are ideal for tissue engineering. Their low cost and commercial availability render them attractive for developing advanced functional biomaterials for gene and drug delivery, cellular regenerative medicine, biosensors, and photothermal therapies [11].

Carbon nanomaterials are ideal for crafting 3D tissue scaffolds, given their compatibility with natural extracellular matrices and remarkable mechanical strength and conductivity. Protein–GO/rGO scaffolds have enhanced mechanical properties due to the G sheets’ high specific surface area and their strong interactions with protein chains. GO derivatives’ dimensions, shape, and surface decoration are crucial for their interactions with biologically active molecules [11].

Photoaging in aquatic environments causes GO to enter biota and interact with proteins. GO may form a protein corona on its surface after interacting with proteins in biological systems. A protein corona is a protein layer that forms when nanoparticles come into contact with biofluids. Protein conformation and biological functions might alter due to such interactions. Understanding the interactions between GO and proteins and the induced changes in protein conformational structures is critical for predicting GO’s biological risks to human health [81]. The physicochemical properties of GO determine protein–GO interactions. GO released into the environment may deteriorate under photoirradiation and change physicochemical properties. Chemical reduction and photoirradiation can form rGO to varying degrees. Therefore, GO and rGO exhibit distinct aggregation behaviors in physiological environments, significantly influencing their interactions with proteins such as those from fetal bovine serum [82]. Research shows that chemically reduced rGO does not disperse as well, consequently having less inhibition of lysozyme activity. In general, smaller nanoparticles with larger surface areas have higher protein affinity. BSA and lysozyme are model proteins due to their prevalence in biological environments and distinct conformational structures, amino-acid residues, and surface charges [83].

Additionally, several other proteins, such as hemoglobin, streptavidin, fibronectin, pepsin, and myoglobin, have been functionalized on GO to leverage their unique properties.

Hemoglobin: Functionalized GO–hemoglobin conjugates have been explored for their oxygen-carrying capabilities, making them potential candidates for artificial blood substitutes and biosensors. Hemoglobin is attached to GO through non-covalent interactions and covalent bonding, ensuring stable conjugation while preserving its oxygen-binding capacity. Studies have shown that GO–hemoglobin composites can enhance the stability and functionality of hemoglobin, making them suitable for biomedical applications, including oxygen delivery and biosensing [66].

Streptavidin: GO–streptavidin conjugates are extensively utilized in biosensing applications due to the robust biotin–streptavidin interaction. Streptavidin is covalently bonded to GO, typically using carbodiimide chemistry to link streptavidin to carboxyl-functionalized GO. Moreover, the strong affinity between streptavidin and biotin (a small, naturally occurring vitamin) allows the conjugates to be further functionalized, significantly improving the sensitivity and specificity of biosensors. This enables the efficient detection of various biomolecules, including nucleic acids, proteins, and small molecules, across various diagnostic assays [67].

Fibronectin: Functionalized GO–fibronectin scaffolds are used in tissue engineering to promote cell adhesion and growth. Fibronectin is covalently attached to GO using linker molecules such as 1-ethyl-3-(3-dimethylaminopropyl)carbodiimide (EDC)/N-hydroxysuccinimide (NHS) to activate carboxyl groups on GO, forming amide bonds with fibronectin’s amino groups. These scaffolds enhance tissue regeneration by providing a conducive environment for cell proliferation and differentiation, making them ideal for wound healing and regenerative medicine applications [84].

Pepsin: GO–pepsin conjugates are investigated for their potential in biocatalysis and developing novel enzymatic systems. Pepsin is immobilized on GO through covalent bonding, typically using glutaraldehyde as a cross-linker. This immobilization preserves pepsin’s enzymatic activity and stability, enabling its use in various industrial and biomedical applications, including protein digestion and pharmaceutical manufacturing [69].

Myoglobin: GO–myoglobin conjugates are studied for their applications in biosensing and bioelectronic devices. Myoglobin is functionalized on GO using non-covalent and covalent interactions, ensuring stable attachment while maintaining its redox properties. These conjugates enhance the electrochemical performance of biosensors, making them suitable for detecting oxygen levels and monitoring physiological conditions in real-time [85].

### 5.3. Interaction Mechanisms

Understanding the interaction between peptides/proteins and GBMs is crucial for diverse biomedical and biotechnological uses, as the functionalization of GBMs by protein or peptide addition can happen through covalent or non-covalent binding [11].

#### 5.3.1. Non-Covalent Interactions

Peptides and proteins attach to GO surfaces through non-covalent interactions, providing an efficient means for scaffold reinforcement. These interactions encompass electrostatic forces, van der Waals forces, hydrophobic interactions, hydrogen bonds, and π–π stacking [11]. Zhang et al. [64] investigated the binding of GO with BSA, uncovering the involvement of hydrophobic forces, hydrogen bonds, van der Waals interactions, and π–π* stacking. Circular dichroism (CD) studies indicated significant alterations in protein and peptide structures in the presence of GO, reducing α-helix content and affecting BSA’s stability against thermal degradation and drug binding. The surface functionalization of GO influences the stability of biologically active molecules. Bai et al. [86] found contrasting effects of GO and rGO on lysozyme, with GO inhibiting activity and inducing structural loss, while rGO maintained activity and increased α-helix content. Bera et al. [87] observed reduced thermal stability in GO-bound lysozyme, noting GO binding at the protein’s active site, reducing activity. Non-covalent functionalization, common in peptide or protein scaffolds for tissue engineering, offers advantageous fabrication but may result in weak stability and reduced protein activity. Conversely, covalent conjugation can enhance stability against various conditions [11].

During the non-covalent binding of peptides/proteins to GO, several interactions may occur between the functional groups of the peptides/proteins and the functional groups present on the surface of GO [88]. These interactions include the following:

- π–π stacking: Aromatic residues in peptides/proteins can interact with the aromatic rings of GO via π–π stacking interactions. For example, melittin, which contains aromatic residues such as phenylalanine, tyrosine, and tryptophan, can engage in π–π stacking with GO, enhancing binding affinity and stability [89].
- Electrostatic interactions: Positively charged amino-acid residues in peptides/proteins can interact with the negatively charged functional groups on the surface of GO. For instance, histone proteins rich in lysine and arginine residues can form electrostatic interactions with the carboxyl and hydroxyl groups on GO [90].
- Hydrophobic interactions: Hydrophobic residues in peptides/proteins can interact with the hydrophobic regions of GO, promoting strong binding. An example is the β-amyloid peptide, which contains hydrophobic residues such as valine, isoleucine, and leucine, facilitating hydrophobic interactions with GO [91].
- Hydrogen bonding: Functional groups capable of hydrogen bonding in peptides/proteins can interact with similar groups on GO. Silk fibroin, with its hydroxyl, carboxyl, and amino groups, can form hydrogen bonds with GO, enhancing the stability of the conjugate [92].
- Van der Waals forces: Non-specific van der Waals forces between peptides/proteins and the surface of GO contribute to binding stability. BSA, for instance, can engage in van der Waals interactions with GO, aiding in forming a stable conjugate [64].

These non-covalent interactions facilitate the binding of peptides/proteins to GO, resulting in functionalized materials with enhanced properties for various applications, including biomedical, biotechnological, and environmental fields [88].

#### 5.3.2. Covalent Interactions

Covalent binding of peptides and proteins to GO/rGO layers improves compatibility and dispersion. This involves strong binding via protein amino acids and modified GO/rGO groups (carboxyl, hydroxyl, or epoxide groups) [11]. The most common approach involves coupling agents like 1-ethyl-3-(3-dimethylaminopropyl)carbodiimide (EDC), and N-hydroxysuccinimide (NHS), activating the carboxyl groups on GO to bind with amino groups on peptides or proteins, thus forming stable amide bonds [93]. Alternatively, the reactive groups on GO can directly interact with amino-acid residues on peptides/proteins, thereby forming covalent bonds. Su et al. [94] enhanced scaffold stability by immobilizing protease on GO with glutaraldehyde. Amine dendrimer-functionalized GO increased BSA stability. Hermanová et al. [95] showed GO-immobilized lipase’s improved resistance to heat and solvents. Despite existing studies, a deeper understanding is needed for biological processes and regenerative healing. Yet, covalent protein–GO scaffolds show promising benefits over non-covalent ones, holding potential for tissue engineering [11].

During the covalent binding of peptides/proteins to GO, several interactions may occur between the functional groups of the peptides/proteins and the functional groups present on the surface of GO. These interactions contribute to the formation of stable chemical bonds [51]. Some of the key interactions involved in covalent binding include the following:

- Amide bond formation is the primary interaction in peptide/protein conjugation with GO. It occurs between the carboxyl groups of the peptides/proteins and the amine or hydroxyl groups on the surface of GO. The reaction typically proceeds via condensation reactions, resulting in the formation of stable amide bonds. For example, LL-37 can form amide bonds through a condensation reaction between its carboxyl group and the amine or hydroxyl groups on GO [96].
- Esterification involves the reaction between carboxyl groups of peptides/proteins and hydroxyl groups on the surface of GO. This reaction leads to the forming of ester bonds, contributing to the covalent attachment of peptides/proteins to GO. For instance, lysozyme can react with the hydroxyl groups on GO to form ester bonds, contributing to the covalent attachment [97].
- Thiol chemistry: thiol groups (-SH) present in peptides/proteins can react with various functional groups on the surface of GO, such as epoxides or carboxyl groups, through thiol-ene or thiol-epoxy reactions. This results in the formation of thioether bonds, which contribute to covalent binding. For example, insulin, known for its structural flexibility and containing cysteine residues with thiol groups, can form thioether bonds with GO [98].
- Click chemistry: click chemistry reactions, such as azide–alkyne cycloaddition (CuAAC) or strain-promoted alkyne–azide cycloaddition (SPAAC), can be utilized for covalent conjugation of peptides/proteins to GO. Functional groups, such as azide or alkyne, are introduced onto peptides/proteins and GO surfaces, enabling selective and efficient covalent binding via click chemistry reactions [99]. CPPs (such as transportan, TAT, penentratin, R8, and MAP) modified with azide or alkyne tags can undergo click chemistry reactions with GO surfaces functionalized with complementary groups [10].

These interactions facilitate the formation of stable covalent bonds between peptides/proteins and GO, forming functionalized materials with enhanced properties for biomedical, biotechnological, and environmental applications [51].

## 6. Utilization of Graphene-Based Materials in the Biomedical Sector

**Figure 4:**
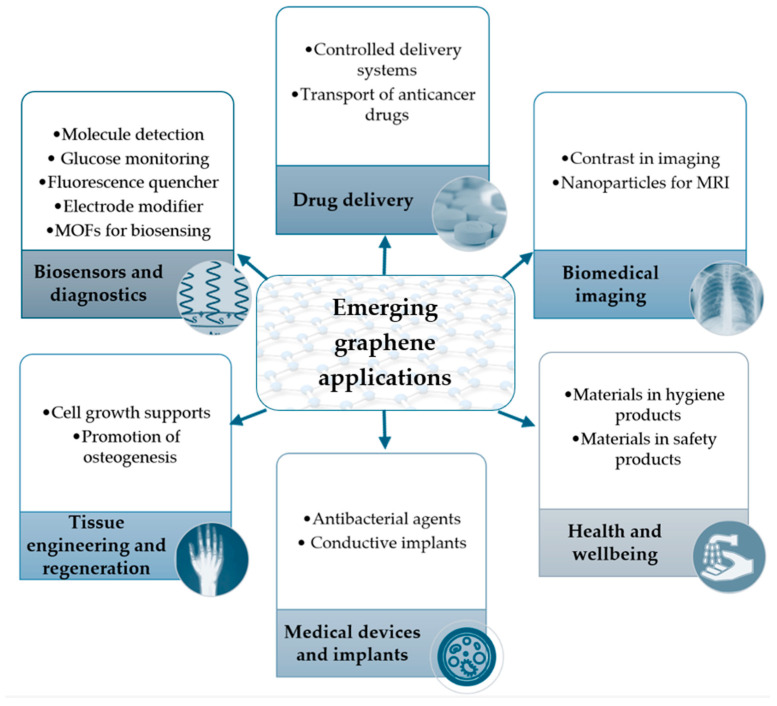
Opportunities for graphene-predicted implementation in biomedical-related sectors [2,108].

As is currently known, G consists of a single layer of carbon atoms arranged in a hexagonal lattice. Although it is a recent development, it exhibits unique physico-chemical and biological properties, making it highly promising in the biomedical sector. These properties enable its potential for a wide range of applications, offering innovative solutions to various medical challenges [100,101,102,103,104,105,106,107]. Figure 4 shows some of the possible implementation areas of this pioneering vision. In this domain, some places with encouraging perspectives vary from screening and different molecule identification to pharmaceutical vectors, recuperation roles, or composite elements. The methods to synthesize the G and its derivative form have evolved. Liu et al., for example, highlighted in their research the possibility of obtaining a targeted dimension of GO or GOQDs only by shifting the ratio of the reagents, respectively, and the reaction temperature [24].

The explicit polyvalence of G in the biomedical field is currently rapidly evolving. The relevant literature studies were the basis for evidencing them in Figure 4 [2,108].

### 6.1. Graphene Used for Biosensors and Diagnoses

Various biosensor designs have emerged using a range of materials, including silica nanoparticles, metal colloids, and advanced engineered materials like molecularly imprinted polymers (MIPs), metal-organic frameworks (MOFs), and quantum dots (QDs). Carbon-based materials such as fullerenes, graphene/graphene oxide, and nanotubes are employed, often in hybrid combinations, to leverage their synergetic properties and address the limitations of traditional biosensors [109].

G is used in ultra-sensitive biosensors to detect extremely low concentrations of biomolecules, such as proteins, DNA, or disease markers [110], including those for various cancers (e.g., breast cancer [111]) or microorganisms [112]. Due to its excellent electrical conductivity, high surface area, and chemical functionalization capability, it can amplify electrical signals generated by molecular interactions with target particles. As a result, the detection of different biomolecules is very accurate. G is chemically modified to increase selectivity. In this way, the specificity of the binding process is improved through background interference reduction. Oxidation (GO) [113] and reduction (rGO) [114] enhance the hydrophobicity and drug loading capacity. 

#### 6.1.1. Graphene Oxide as a Fluorescence Quencher in Biosensors

GO is considered a fluorescence quencher, making it highly suitable for applications like fluorescence resonance energy transfer (FRET). FRET is widely used for detecting targets and studying molecular interactions, where GO’s quenching properties are critical [115]. In this regard, Zeng et al. [116] present sST2 as a novel biomarker for heart failure diagnosis and prognosis. They developed a GO-based FRET biosensor using the aptamer L1, which achieved a detection limit of 3.7 ng/mL for sST2. This biosensor strongly correlated with traditional ELISA in serum samples from heart failure patients, providing a rapid and sensitive detection method. 

In a related advancement, Huang et al. proposed a highly advanced point-of-care biosensor that utilizes fluorescence quenching to quantify various cancer biomarkers, including gene p53 and prostate-specific antigen (PSA). This biosensor leverages GO’s fluorescence-quenching properties to offer precise and sensitive detection of these biomarkers. Incorporating GO enhances the sensor’s performance by improving sensitivity and specificity, addressing limitations observed in conventional detection methods [117].

These developments underscore the growing potential of GO-based fluorescence-quenching techniques in biosensor applications. While Zeng et al.’s GO-based FRET biosensor [116] provides a practical solution for heart failure diagnosis, Huang et al.’s [117] point-of-care biosensor extends the application of fluorescence quenching to cancer biomarker detection, illustrating the versatility and impact of GO in advancing biosensor technology.

#### 6.1.2. Graphene/Graphene Oxide as an Electrode Modifier for Peptide Functionalization

Heavy metals, while essential in small amounts (e.g., manganese and copper), become harmful at higher concentrations, necessitating fast and sensitive detection methods [118]. Bagheri and colleagues [119] developed an electrochemical sensor using a graphene-based composite to detect Tl^+^, Pb^2+^, and Hg^2+^ in various samples without pre-separation. Similarly, gold nanoparticles and reduced graphene oxide were used to modify electrodes for detecting Cu^2+^, achieving high sensitivity in soil samples [120]. Other studies [121,122,123] have applied graphene-based sensors to detecting heavy metals such as thallium, lead, cadmium, and copper in environmental and food samples, showing strong agreement with conventional methods. Various graphene composites, including those combined with polyaniline, Bi2Te3, and N-doped graphene, have further enhanced detection sensitivity for trace metal ions in water, soil, and food products [124]. These advanced electrochemical platforms offer promising solutions for environmental monitoring and food safety [118].

#### 6.1.3. Enhancing Metal–Organic Frameworks with Graphene Materials for Advanced Biosensing Applications

Metal–Organic Frameworks (MOFs) are crystalline materials composed of metal ions or clusters linked by organic ligands to form a porous three-dimensional network. They are characterized by their high surface areas, tunable pore sizes, and adjustable chemical functionalities, making them valuable for various applications, including gas storage, separation, catalysis, and sensing. When combined with graphene oxide (GO) or other graphene materials, MOFs benefit from improved electrical conductivity, enhanced stability, and prevention of particle aggregation. These enhancements also lead to increased surface area and active sites, significantly boosting the performance of MOFs in applications such as biosensors. The integration of graphene materials enables MOFs to achieve higher sensitivity and efficiency in detecting biomolecules and metal ions, making them highly effective for advanced biosensing applications [125,126].

Tang et al. developed an electrochemical sandwich-type immunosensor for detecting galectin-3 (Gal-3), leveraging Au@MIL-88(Fe)-NH_2_ functionalized GCE electrodes. They synthesized the MOF from FeCl_3_ and 2ATPA, combined it with HAuCl_4_, and reduced it with NaBH_4_ to create AuNPs@MOF, further enhanced with nitrogen-doped graphene nanoribbons (N-GNRs). This biosensor achieved a detection range of 100 fg/mL to 50 ng/mL and a limit of detection (LOD) of 33.33 fg/mL, with high reproducibility and excellent recovery in spiked serum samples [127].

In a parallel approach, Yu et al. developed an electrochemical biosensor for Pb^2+^ detection using the MOF MIL-88(Fe)-NH_2_ combined with platinum–palladium nanoparticles (PtPdNPs) created by reducing metal salts with NaBH_4_. The biosensor featured a graphene-based electrode functionalized with AuNPs@N-doped graphene sheets. This setup achieved a limit of detection (LOD) of 0.003 fM and was tested on spiked serum and human blood samples. In a related study, the same MOF was used in a biosensor for miRNA-122 quantification, which utilized graphene for signal enhancement, achieving a detection range from 0.01 fM to 10 pM [126].

Both studies exemplify how combining MOFs with advanced materials like graphene and metal nanoparticles can significantly enhance the sensitivity and applicability of electrochemical biosensors, addressing different detection needs, from biomarkers to metal ions.

Table 2 summarizes some of the newest results in G-based biosensors for biomedicine, underling the specific detection limit.

The development of integrated G-based sensors exhibits promising features. Designing devices based on this technology requires considering portability and flexibility [135]. In this way, the parameter’s status is tracked in real-time.

A particular focus was developed around fluorescent biosensors with satisfactory selectiveness and responsitivity. Good results have been obtained in targeting mainly native proteic biomolecules [136] or substituted [137]. Wang et al. underlined the possibility of implementing such an idea for protein kinase activity recognition [138].

### 6.2. Graphene’s Utility in Drug Delivery

Graphene and its functionalized derivatives, such as GO and rGO, can be used to create controlled drug-delivery systems that deliver active substances precisely at the target site. Their effectiveness is attributed to water-processing capacity, amphotericity, and luminescence suppression [2]. Such an approach reduces side effects and improves the efficacy of treatments. G’s ability to load and release anticancer drugs into specific tumor cells makes it a valuable tool in oncology treatments. Its unique properties also enable the increased transport of various medications. Additionally, these systems overcome some limitations of previous methods by allowing for the extended and precise release of active substances [139].

Moreover, the graphene surface can be engineered to respond to specific physical–chemical stimuli such as temperature, conductivity [140], pH, and/or magnetic fields. This allows for the development of drug-delivery systems that release drugs in a controlled manner, further enhancing their precision and responsiveness. In their study, Mianehrow et al. highlighted the advantages of incorporating GO in the drug vector containing chitosan and hydroxyethyl cellulose. G addition balanced the system behavior, making it suitable in all pH ranges [141].

Through biofunctionalization with target molecules such as antibodies, peptides, or other recognition molecules, a drug carrier that specifically recognizes and binds to diseased cells, such as cancer cells, can be obtained. Such an approach reduces the side effects and increases the effectiveness of the treatment. Karimi et al. successfully tested the behavior of the GO-TD-Fe_3_O_4_@PEG nanocomposite response to pH variation. The focus was on the composite capabilities to discharge the antineoplastic material as intended [142]. The G-based yolk–shell magnetic nanoparticles (GYSMNP) functionalized with copolymer PF-127 tested by Rodrigues et al. that exhibited hydrophilic activity proved to be beside medicine vectors also heating capacities [143]. The potential of the superparamagnetic iron oxide-reduced GO (Fe_3_O_4_-RGO) nanohybrid studied by Gupta et al. was proved against human cervical cancer cell lines [144]. Maximum efficiency was observed in the case of magnetic field-assisted hyperthermia.

It was proved that G can penetrate cell membranes and carry drugs directly inside them. This ability is essential for treating certain diseases that require intracellular drug delivery. The GO, Fe_3_O_4_ nanoparticles, and lactoferrin system exhibited good cytotoxic behavior over C6 glioma cells. The complex was pH-dependent and had an increased utility surface [101].

In combination with imaging contrast agents (gold nanoparticles or MRI agents), G could contribute to developing multifunctional platforms that deliver drugs and enable visual monitoring of treatment distribution and efficacy. Kumara et al.’s in vitro results for their carrier systems based on lysozyme, chitosan, and G quantum dots (GQD) validate the possibility of passing further to the preclinical stage [145].

Different studies have shown that G and its derivatives can be modified to be biocompatible [146] and have adequate bioavailability. Jariya et al. proved the vaterite–rGO/PCL (where PCL is a polycaprolactone matrix) composite coating potential to deliver constant antibiotic (ciprofloxacin) release. The system was also designed to have good biocompatibility and bioavailability [147]. By functionalizing the G surface, toxicity [100] and side effects can be reduced, thus improving the safety profile of the drug carrier. Tabish et al. highlight the possible use of S-nitroso cysteamine-functionalized porous GO nanosheets as carriers for NO, a marking molecule linked to cardiovascular disease. Their in vitro promising research results could be the starting point for implementing such a system [148].

After functionalization, G can carry chemotherapeutic drugs directly to cancerous tumors [149,150,151], reducing damage to healthy tissues [152]. The same pattern was observed also in the case of antibiotic administration. In this case, the effectiveness of treatment is enhanced, and the bacterial resistance is reduced. Promising results were also observed when G delivered genetic material such as DNA or RNA. These were integrated into gene therapies to correct genetic defects or modulate gene expression [153].

### 6.3. Graphene Application for Biomedical Imaging Purposes

**Figure 5:**
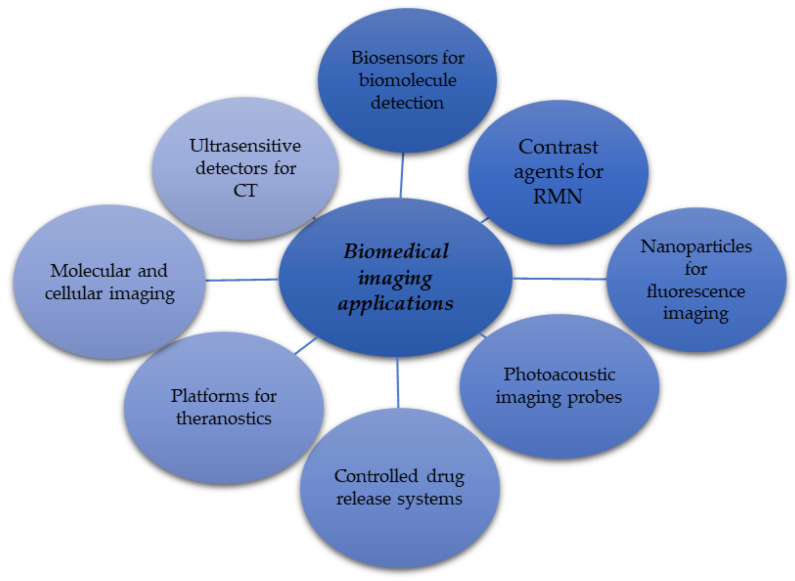
Developed applications of graphene in the biomedical imaging field.

Figure 5 briefly summarizes the possible development directions for implementing G in the imagistic area. Its application in this domain is an emerging field that promises to revolutionize how medical diagnoses and treatments are made. G’s exceptional properties, such as electrical and thermal conductivity, mechanical strength, and high specific surface area, make it ideal for various imaging applications. Before preparing their particular usage, G is functionalized to enhance imaging techniques such as magnetic resonance (MRI) or fluorescence imaging. In this way, the visualization of tissues and cellular structures becomes more precise. Together with iron nanoparticles, they are more effective contrast agents in MRI than commercially available agents [154]. Figure 5 highlights the comprehensive areas of possible applications developed only for the imaging field. The many studies in the specific literature comprised the foundation for schematically outlining the currently proposed and studied implementation fields [155,156,157].

G and its derivatives, such as GO [158], could serve as contrast agents for MRI. Torkashvand and Sarlak proved the potential of the GO-PCA/MnCe_0.5_Fe_1.5_O_4_ nanofluid obtained by co-precipitation to be used as a contrasting factor for magnetic nuclear resonance for biotic structures [159].

Due to their ability to influence magnetic signals, these agents can significantly improve the clarity and resolution of MRI images. G can be functionalized with various chemical groups, such as the methyl group [158], to specifically target specific cells or tissues, thus facilitating the accurate diagnosis of diseases.

Li et al. successfully developed a SARS-CoV-2 detection system containing G quantum dots (GQDs)-based magnetic relaxation switch (MRSw). The method presents several advantages compared with the current alternatives. The time for the operation is shortened since there is no need for pretreatment, presents an increased biological safety level because the samples are not reopened, and is feasible for implementation in cases of other viral material identification through Nuclear Magnetic Resonance (NMR) relaxometry methodologies by simple antibody replacement [160].

G can be used to obtain fluorescent nanoparticles and for in vitro and in vivo fluorescence imaging. These nanoparticles have unique optical properties that help label and track cells in real-time, providing valuable information about cellular dynamics and disease progression. The system containing polyethylene glycol-modified GO quantum dots and folic acid may have good applicability for MRI fluorescence to diagnose neoplasms. Besides the significant yield of cell absorption, the method also proved to have low toxic effects [161]. Table 3 includes recent research results from the scientific literature concerning the system projected to improve G attributes in fluorescence imaging.

Today’s medicine also faces the problem of finding non-invasive or minimally invasive solutions for one of the diseases of the century, namely the different types of cancer. The current trend is to find ways to spot carcinomas and, as far as possible, to neutralize [167] them using a single medical device that is efficient, selective, and has limited negative effects on adjacent tissues or the body. It seems that medical instruments that incorporate G or its derivatives can respond to these wishes. Developing systems that can act synergistically through at least two mechanisms can effectively respond to such projections [168]. Some researchers reported similar activation elements for drug releases, such as pH [169,170] or off-site NIR light [169].

Chang et al. showed the possible potentiation of specific effects in the case of the GO/MnWO4/PEG system. The in vitro results were sustained by the in vivo one. The complex had photothermal–chemotherapy capacities [169].

G can be used in photoacoustic imaging probes, which combine optical and acoustic imaging advantages. G has excellent light absorption and can generate acoustic, solid signals when illuminated, enabling high-resolution imaging of deep biological structures such as blood vessels and tumors.

Sheng et al. describe the promising benefits of their protein-based system containing reduced GO (nano-rGO). The designed systems tested in mice tumors presented good photoacoustic/ultrasonic and photothermal properties, stability, and decreased toxic effects [171].

As mentioned previously, G can be used to develop controlled drug release systems complementary to imagistic monitoring. G nanoparticles can carry drugs directly to target tissues and can be tracked by imaging techniques, thus ensuring that drugs are released in a controlled and efficient manner. Table 4 comprises some of the systems projected in this regard.

G can be used in theranostic imaging platforms, which combine diagnostics and therapy in a single system. These can deliver drugs directly to the identified disease site through a noninvasive mechanism [180] and monitor treatment response in real-time using imaging techniques such as MRI or photoacoustic imaging. Such an approach allows the personalization of treatments and the improvement of therapeutic efficiency, responding in this way to two major inconveniences of the classical carcinoma treatment challenges, namely the multiple side effects and precision deficiencies [181]. One of the currently appreciated theranostic systems incorporates, besides G dots, different peptides that exhibit promising benefits [181,182]. Fateh et al. developed a complex platform with minimal cell toxicity, fluorescence, and marking capacities. The surfactant properties of the GQDs were obtained through pyrolysis in the presence of cetyl alcohol (CAGQDs). The structure was completed with iron-oxide nanoparticles [183]. Saranya et al. propose a hybrid nano platform containing CeO_2_/Au/GO in their study. It could be suitable for cervical cancer investigations and treatment [184].

G binds to specific molecules through functionalization, enabling detailed molecular and cellular imaging. Such applicability is crucial for cancer research, where identifying and visualizing tumor cells at the molecular level can lead to more accurate diagnoses and the development of targeted therapies. Bugárová et al. designed a pattern network containing GO and biotinylated M75 antibodies that could have possible applications in carcinoma treatment strategies [185].

### 6.4. Graphene Use in Tissue Engineering and Regeneration

G can be utilized to create biocompatible supports that stimulate cell growth, ensuring their physical support. Its characteristics make it ideal for tissue regeneration or even organ engineering. G-based supports have demonstrated efficacy in stimulating bone formation and soft tissue self-reconstruction [186], which is valuable in treating fractures or orthopedic implants. It can be used to obtain scaffolds that support cell growth and proliferation. These scaffolds could mimic natural tissue’s structure and function, facilitating the regeneration of bone, muscle, blood vessels [187], or nerve tissue with particular application for brain structure reconstruction. Finding solutions in this area could bring enormous benefits because, for a long time, this type of cell was considered to be non-regenerative. Ghosh et al. sustained in their study the positive benefits of their chitosan fine-coating devices strengthened with gold nanoparticles (GNPs) [188]. The particularities are determined by its potential to conduct electricity, contributing simultaneously to improving cell adhesion and proliferation due to its large surface area and chemical properties, which are crucial for integrating scaffolds into native tissues. Among the most critical aspects that must be followed in the case of such medical devices are their biocompatibility and the degree of cytotoxicity [189]. In bone tissue reconstruction, other significant properties are mechanical attributes. Also, G-based composites have proven to influence their improvement positively. The poly-3 hydroxybutyrate–chitosan and GO structure is a proposed and tested solution [190].

An aspect that has to be highlighted refers to the source of the primordial cells used. In several current results, the source of raw material was biotic. Promising results have been obtained by Wang et al., which technology was based on a laser-induced mechanism. As a carbon source, they considered bone collagen [191]. As possible peripheral nerve reconstruction, Zaman et al. propose the *Lisianthus* flower stems [192]. Verstappen et al. developed their nanocomposite around a porcine adipose tissue-derived extracellular pattern and rGO [189]. Considering the proportion of today’s necessities in bone reconstruction, it could also be essential to consider the possibility of implementing bio-economy principles in this area. A possible solution was given by Sampath et al. Their research was conducted around the idea of using hydroxyapatite obtained from seashells. The proposed solution contained hydroxyapatite–GO–chitosan–carbon nanotube–polylactic acid and exhibited promising features, including drug delivery and nanomedicine [193].

Although the specific literature reports most of the possible applications for bone regeneration, Meira et al. researched the zone of G-based composite for cardiac tissue reconstruction. Their G/P(VDF-TrFE) coating structures showed good biocompatibility [194]. Promising results have also been obtained by Kaviani et al. Their GelMA/Alginate/Polypyrrole/G structure was initiated as a hydrogel. Two of the characteristics that have to be mentioned refer to remarkable sanguine compliance and versatility [195].

### 6.5. Graphene Applications as Medical Devices and Implants Based on Its Antimicrobial Properties

G has antibacterial attributes that may be useful in developing medical devices and implants that reduce the risk of infection. It is well known that any surgical intervention also presents an infection risk. The hazard increases in specific situations, such as insertion in the body of different exogenous devices at which the microorganism could colonize the surface. Usually, the devices have a metal structure that might conduct body rejection. Solutions to such situations could come from developing systems containing G and collagen [196] or polydopamine, silver or tetracycline hydrochloride, and oxidized G. Promising perspectives were obtained based on the last combination against *Escherichia coli* and *Staphilococcus aureus* [197]. Recent studies highlight the potential use of G and/or its derivatives in systems with coating [198] and antimicrobial properties. Romo-Rico et al. analyzed the effect of G installment on the medical-grade cobalt–chromium (CoCr) metal side. Firstly, the essential oil of *Origanum vulgare* was considered and successfully used as a resource for volatile carbon to give the desired G. The antibacterial activity of the G and its derivative was tested against *Pseudomonas aeruginosa* and *Staphylococcus aureus*. Promising results were obtained in both cases [199]. A potentiation effect was observed in the case of GO and gallium nanoparticles concerning their antibacterial and osteogenic potential [200].

In any case of implants, it pursues the device’s mechanical strength. Qin et al. highlighted the possible relevance of the composite they designed. The complex consisted of graphite oxide that enhanced the carbon fiber interface. These were strengthened with poly–ether–ether–ketone (PEEK). The suggested applications of the PEEK/SiO_2_/GO composites targeted the orthopedic and dental areas [201]. In the same field of medical applications, composites obtained based on G and calcium phosphate (hydroxyapatite) could also be considered composites [198].

Due to its electrical conductivity, G can be used to create smart implants, such as electrodes for nerve stimulation or for monitoring brain activity. The biochemical generator tested by Menassol et al. proved to have possible in vivo applicabilities. It consisted of a system incorporating iron/nitrogen co-doped reduced GO-based electrodes as a cathode. The observations made in the presence of fibroblast cells sustain the reliability of use and biocompatibility for a more extended period [202].

G can be used in personal hygiene products such as face masks or air filters due to its antibacterial properties and ability to filter fine particles. Table 5 summarizes some recently tested applications of G-based composites that could be implemented in the biochemistry and microbiology fields. One advantage of detection systems incorporating GO is the possibility of parallel detection of interest analyses. An example is the multilayer system developed by Zhao et al. [203]. Such an approach proved to be sensitive. The time reduction for the detection could also be mentioned since the procedures are developed simultaneously by a single operator and not consecutively, as well as the reduction of chemicals involved in the classical detection and quantification procedures.

## 7. Limitations

The research encompasses a diverse array of subjects, ranging from synthesis techniques to biological uses. However, due to this extensive scope, many areas may not have received the same level of thorough investigation as others. Furthermore, much of the research predominantly emphasizes well-established methodologies and applications, neglecting this sector’s newer, developing approaches and fast-evolving discoveries.

While the studies examine the potential uses of GBMs, they often fail to thoroughly address long-term consequences, particularly regarding toxicity and environmental effects. The use and manufacture of materials based on graphene might result in noteworthy environmental ramifications that necessitate meticulous deliberation. For example, creating graphene oxide and its derivatives requires potent oxidizing agents and other chemicals. Improper management of these substances can result in the production of dangerous waste and the risk of contamination. Moreover, the whole lifespan of these materials, starting from their manufacturing and ending with their disposal, presents significant sustainability and environmental safety obstacles. The long-term presence of graphene materials in the environment and their interactions with ecosystems are still not comprehensively known, which gives rise to worries over the possible buildup of these materials in living organisms and the resulting impacts on wildlife and human well-being.

Furthermore, there is a need for more extensive research on the toxicity of graphene-based materials, both in the short and long term. Although certain types of graphene and its derivatives have been shown to have minimal toxicity in certain studies, other studies indicate that these materials can have adverse biological consequences, especially when they come into contact with cellular membranes or accumulate in organs. The possibility of long-term exposure, mainly due to the growing use of these substances in different applications, may result in unexpected health hazards. In addition, the variations in the characteristics of graphene materials, such as their size, surface chemistry, and functionalization, can lead to considerable differences in their toxicity. 

## 8. Conclusions and Perspectives

Graphene-based materials (GBMs) have extensive promise in numerous disciplines, especially in biological applications, as emphasized by this study. Nevertheless, several viewpoints arise from the constraints above, which might provide direction for further investigation and advancement. An essential view requires more extensive research that achieves a harmonious combination of thoroughness and inclusiveness. Although the present studies offer a comprehensive overview, future research should focus on exploring specific areas, such as the intricate mechanics of graphene’s interaction with biological systems or the potential long-term consequences of its application in therapeutic settings.

Furthermore, the rapidly advancing methodologies and innovative uses of GBMs necessitate a more thorough investigation. We must continually enhance our knowledge and stay up to date with technical progress as we create new techniques for synthesizing and functionalizing graphene. This involves investigating the most recent advancements in green synthesis techniques, which can potentially reduce the environmental consequences emphasized in this research.

Future research should focus on developing standardized testing methods and regulatory frameworks to ensure graphene materials’ safe and sustainable use, particularly regarding toxicity and environmental effects. This will necessitate multidisciplinary collaboration, combining knowledge from materials science, toxicology, environmental science, and regulatory authorities.

Furthermore, additional in vivo investigations and clinical trials are required to gain a more comprehensive understanding of the practical consequences of GBMs in medicinal applications. As the area advances, translating laboratory research into useful clinical treatments will be a primary emphasis. This encompasses the assurance of the safety and effectiveness of treatments and diagnostics based on graphene and the resolution of the economic and scalability obstacles associated with their manufacturing and broad use.

By focusing on these specific areas, the field of graphene-based materials may make further progress, discovering new opportunities while ensuring that the advantages are achieved in a secure, environmentally friendly, and meaningful manner.

## Figures and Tables

**Table 2:** Graphene-based biosensors sensing limits.

Sensor Surface Type	Biomolecule Detected	LOD	References
polystyrene	miRNA-21	1.74 pM	[113]
metal–organic frameworks (CuMOF, Fe@rGO)	microRNA 155	0.08 fM	[111]
micro-tapered long-period fiber grating functionalized by graphene oxide (GO-MTLPFG)	pepsin	25.79 ng/mL	[69]
RGO, Au nanoparticles, and the aptamer conjugated on the polythiophene (PTP) modified electrode	*H. pylori* (Hsp60)	0.0080 μM	[114]
printed electrochemical biosensor	glucose	1.34 mM	[128]
six pairs of interdigital Cr/Au electrodes supported on Si/SiO_2_ substrate with an avidin immobilized	biotin	90 fg/mL (0.37 pM)	[129]
aptamer and oligonucleotide–gold nanoparticles	insulin	1.6 × 10^−12^ moL/L	[130]
thiophenol moieties	hepatitis C virus core protein	90.9 aM	[131]
aptamers-functionalized graphene and magnetic β-cyclodextrin polymers	adenosine	2.1 × 10^−13^ mol/L	[132]
(His)-tagged acetylcholinesterase (AChE)	paraoxon	3 μM	[133]
graphene–peptide conjugates	leucine-rich alpha-2 glycoprotein-1	75 pg/mL	[134]

**Table 3:** Recent advancements in graphene-based systems for fluorescence imaging.

Complex Structure	Application	Specifications	References
MMC–Graphene@BODIPY–mPEG (MGBP)	fluorescence and photothermal imaging photo-chemotherapy	complementary has the ability for drug release	[162]
AuNPs/Gn/GQDs	fluorescence and SERS	minimal cytotoxic level in A549 cells	[163]
nitrogen-doped graphene quantum dots (N-GQDs) and gadolinium ions (Gd^3+)^	fluorescence and magnetic resonance imaging	biological imaging and diagnosis, drug release, good colloidal stability, hydrophilic	[164]
MNP–Herceptin–SK–BR3 cell-Herceptin–GQNP	fluorescence immunosensor	HER2^+^ breast cancer cell target	[103]
GQDs and Ag@AuNPs	antibody fluorescence immunoassay	good selectivity and sensitivity for 2019-nCoV mAb	[165]
graphene oxide–silver nanoparticles (GO-AgNPs)	fluorescence fluoresced to DNA	hydro solubility	[166]

**Table 4:** Graphene-based drug delivery systems with integrated imaging capabilities.

Platform Structure	Specifications	References
graphene oxide encapsulated with folic acid-conjugated chitosan	pH sensitive, optimum value 5.3	[172]
Hydroxypropyl-β-cyclodextrins (HP-β-CD) and carboxylated graphene nanomaterial	hydrophilic properties	[173]
GO nanocarrier	acidic environment activation	[174]
furan-containing copolymers embedded with rGO conjugated with maleimide-containing target molecules	photothermal activation	[139]
mGO@AL-g-PHPM@ICG/EP	light stability and photothermal conversion capacity	[175]
graphene oxide/carboxymethylated chitosan (GO/CMCh) nanocomposite hydrogel	minimal hemolysis ratio	[176]
quercetin/lurbinectedin-loaded GO NPs	toxic effect in neoplasm cell	[177]
hyaluronic acid (HA), graphene quantum dots (GQDs), and polyethyleneimine (PEI)	dual drug release system	[178]
graphene quantum dot	antimlaria system (*Plasmodium falciparum*)	[102]
SiO_2_@Fe_3_O_4_-HA-MIL100 and SiO_2_@Fe_3_O_4_-HA-MIL-100-GQDs nanocomposites	antioxidant properties	[179]

**Table 5:** Possible applications in the biomedical field.

Sensor Surface Type	Biomolecule Detected	LOD	References
palladium-modified graphene oxide	zearalenone	0.16 pg mL^−1^	[3]
molecularly imprinted polydopamine films	*Pseudomonas aeruginosa*	1.85 CFU/mL	[204]
sensitive film of Au-nanoparticles furnished with graphene	carcinoembryonic antigen	2.46 MHz/log (ng/mL)	[205]
graphene PVC film	ion-selective	0.1–100 mM	[206]
graphene oxide-Au surface-enhanced Raman scattering tag	*Escherichia coli*, *Staphylococcus aureus*	10 cfu/mL	[203]
